# Expression profiles of the selected genes in tumor and surgical margin in oral cavity cancer

**DOI:** 10.1186/1897-4287-13-S2-A13

**Published:** 2015-11-26

**Authors:** Joanna Katarzyna Strzelczyk, Karolina Gołąbek, Łukasz Krakowczyk, Aleksander J Owczarek, Martyna Fronczek, Andrzej Wiczkowski

**Affiliations:** 1 Chair and Department of Medical and Molecular Biology, School of Medicine with the Division of Dentistry, Medical University of Silesia in Katowice, Zabrze, Poland; 2 Clinic of Oncological and Reconstructive Surgery, Maria Sklodowska-Curie Memorial Cancer Center and Institute of Oncology, Gliwice Branch, Gliwice, Poland; 3 Statistical Division, Department of Instrumental Analysis, Medical University of Silesia in Katowice, Poland

## 

In the past years, the number of anew diagnosed patients with head and neck squamous cell carcinoma (HNSCC) is still growing. Generally, HNSCC proliferates fast, is locally aggressive and has a high tendency to metastasize to cervical lymph nodes. Head and neck cancer is associated with gene-environment interaction. Several genetic alterations have been reported to contribute to the development of HNSCC, while cigarette smoking, alcohol drinking, chronic exposure to certain industrial agents and infection with specific subtypes of human papillomaviruses (HPV) are considered to be important risk factors, too.

Primary HNSCC tumors and matching surgical margin samples were collected following surgical resection from 56 patients in the Clinic of Oncological and Reconstructive Surgery, Maria Sklodowska-Curie Memorial Cancer Center and Institute of Oncology, Gliwice. Their medical records provided necessary medical and demographic data including patient's age, gender, tumor grade and stage, regional lymph nodes, cigarette smoking and alcohol drinking. The Ethics Committee of Maria Sklodowska-Curie Memorial Cancer Center and Institute of Oncology in Gliwice approved the study protocol (No KB/493-15/08 and KB/430-47/13). Average age of the patients was 56.05 (range: 29-77 years, median 58.5 years). The study included 37 males and 19 females. 80% of patients were smokers, 73% of them reported alcohol consumption, and 64% were both smoking and alcohol drinking. Obtained paired non-malignant tissues were situated over 10 mm from the tumor lesion margins and were histologically confirmed to be cancer-free. The aim of the study was to analyze the significance of *p16, APC, MGMT, TIMP3, SFRP1, SFRP2, CDH1, RASSF1, RORA, DAPK1 *genes expression in tumors and matching surgical margin samples. Total RNA was extracted with RNeasy Mini Kit (Qiagen, Germany) from frozen tissue samples. Isolated RNA; after being verified for integrity, quantity and purity; was used for cDNA synthesis (High Capacity cDNA Reverse Transcription Kit with RNase Inhibitor; Applied Biosystems, USA). Gene expression levels for the above mentioned genes were analyzed by quantitative reverse transcription (qRT)-PCR in tumors and matching surgical margin samples. Reaction was performed using specific TaqMan Gene Expression Assays and TaqMan Gene Expression Master Mix in a 7300 Real Time PCR System (Applied Biosystems, USA). The glyceraldehyde-3-phosphate dehydrogenase (GAPDH) gene was used as an internal control. Relative expression levels were calculated according to the 2^-ΔΔCT ^formula.

Two-sample parametrical Student's t-test for independent samples with equal/unequal variances showed statistically significant lower expression of *SFRP1 *in tumor compared to margin samples (0.30 vs 0.62, p < 0.01). One-way analysis of variance indicated a significant main effect of the grading stage (p < 0.05). NIR post-hoc test showed, that grade G3 patients have significantly higher values of *DAPK *gene expression than the G1 group (p < 0.05), as well as G2 (p < 0.01). No association was found between the genes expression and clinical parameters, except *MGMT*, which low expression was associated with smoking (0.87 vs 1.34, p = 0.065) and *DAPK1*, which low expression was associated with alcohol consumption (0.85 vs 1.97, p = 0.074).

It has been known that the survivors of head and neck cancer presented increased morbidity and mortality and second primary tumors are the main reason of mortality in this group of patients. Second primary tumors or local recurrence are thought to be the consequences of "field cancerization" and could be the results of exposure to carcinogens, frequently involving the development of multiple premalignant and malignant lesions. The initial step of field effect is associated with various molecular lesions, e.g. gene expression.

In conclusion, an important implication is that field often remains after surgery and the remaining peripheral tissue (surgical margin) poses an increased risk of developing cancer. Diagnosis and treatment of cancers ought not to concentrate only on the tumor itself, but also on the cancer field effect.

